# A High-Precision Real-Time Pose Measurement Method for the Primary Lens of Large Aperture Space Telescope Based on Laser Ranging

**DOI:** 10.3390/s23104833

**Published:** 2023-05-17

**Authors:** Heng Shi, Junfeng Du, Lihua Wang, Jiang Bian, Guohan Gao, Dun Liu, Bin Fan, Hu Yang

**Affiliations:** Institute of Optics and Electronics, Chinese Academy of Sciences, Chengdu 610209, China

**Keywords:** pose measurement, laser ranging, space telescope, high-precision

## Abstract

The aperture of space telescopes increases with their required resolution, and the transmission optical systems with long focal length and diffractive primary lens are becoming increasingly popular. In space, the changes in the pose of the primary lens relative to the rear lens group have a significant impact on the imaging performance of the telescope system. The measurement of the pose of the primary lens in real-time and with high-precision is one of the important techniques for a space telescope. In this paper, a high-precision real-time pose measurement method for the primary lens of a space telescope in orbit based on laser ranging is proposed, and a verification system is established. The pose change of the telescope’s primary lens can be easily calculated through six high-precision laser distance changes. The measurement system can be installed freely, which solves the problems of complex system structure and low measurement accuracy in traditional pose measurement techniques. Analysis and experiments show that this method can accurately obtain the pose of the primary lens in real-time. The rotation error of the measurement system is 2 × 10^−5^ degrees (0.072 arcsecs), and the translation error is 0.2 μm. This study will provide a scientific basis for high-quality imaging of a space telescope.

## 1. Introduction

A membrane optical telescope is a new transmission-type imaging space telescope based on special functional materials [1,2]. Theoretically, it overturns the traditional imaging systems, which rely on the primary lens with a curved surface. Technically, it breaks through the bottleneck of increasing the aperture, such as telescope weight, tolerance control of primary lens surface, and envelope size of primary lens [3,4]. Similar projects, such as the Membrane Optic Imager Real-Time Exploitation (MOIRE) program supported by US Defense Advanced Research Projects Agency (DARPA) [5,6,7,8], have been called the 21st-century space disruptive imaging technology.

In space, the relative pose (positions and orientations) of the optical elements, especially between the primary lens and the rear lens group, will change due to the changes in the thermal environment and the structure, which will seriously affect the imaging quality of the telescope [9,10,11]. Furthermore, it is found that the tilt and decentration of the primary lens will make the telescope imaging quality decrease obviously [12,13]. Therefore, the pose measurement of the telescope primary lens has become difficult to overcome urgently in membrane optical imaging technology [14,15].

Traditional pose measurement techniques usually adopt multiple sets of measuring equipment and sensors, which are complicated in structure and require high installation accuracy [16,17,18]. It is difficult to meet the pose measurement requirements of the primary lens of a large aperture transmission space telescope, and the calculation process is relatively cumbersome [19,20,21]. Due to excessive system devices and installation errors, more errors will be introduced [22,23].

This paper presents a high-precision real-time measurement method for the pose of the primary lens of a telescope based on laser ranging. First, the high-precision distance information can be obtained using the laser ranging method, and then the high-precision pose information of the primary lens can be obtained by calculation. Finally, the pose of the primary lens can be calculated by using six distances obtained by laser ranging, and the measurement system can be installed freely, which solves the problems of complex system structure and low measurement accuracy in traditional pose measurement techniques. The system has the advantages of a simple structure, convenient installation, and high measurement accuracy.

## 2. Telescope System Design

**Figure 1:**
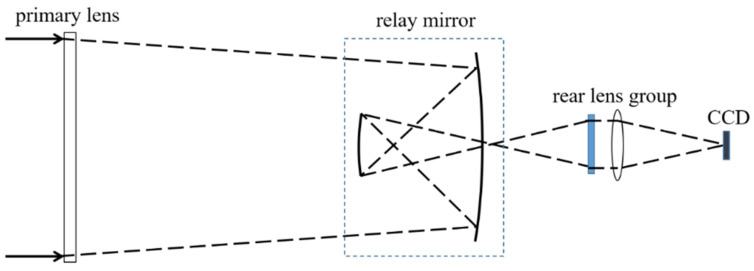
Schematic diagram of the telescope optical system.

We take a long focal length diffraction imaging optical system as an example; the schematic diagram of the telescope optical system is shown in Figure 1. The diameter of the primary lens is 1 m, the primary lens is a plane, and there are annular microstructures prepared on the surface so that the light can converge on the relay mirror. The relay mirror is 300 mm in diameter, and the relay mirror and the rear lens group are fixed in a frame. The relay mirror is used to fold the optical path and shorten the total length of the system. The rear lens group is responsible for correcting aberrations and providing the final image. The primary lens is extended to the right place by trusses, and the distance between the primary lens and the relay mirror is about 3 m. In order to ensure the imaging quality of the telescope, the Modulation Transfer Function (MTF) @62.5 lp/mm should be up to 0.1. To meet the MTF requirement, the system needs to have good optical quality in terms of resolution, contrast, and uniformity. The MTF is a measure of the ability of the system to transfer contrast from the object to the image, and it depends on many factors, such as the aperture size, aberrations, and diffraction effects, which is a challenging requirement for a system with such a long focal length. Defocus tolerance is important for maintaining the focus of the system over time and under different environmental conditions. The focal depth is proportional to the square of the aperture diameter and the wavelength and inversely proportional to the focal length. Therefore, the defocus of the system should be less than 1/4 focal depth during the whole life cycle [24]. In summary, the long focal length diffraction imaging optical system requires careful design and manufacturing to achieve high MTF and low defocus tolerance. The use of annular microstructures, relay mirrors, and rear lens groups are effective ways to improve the optical performance of the system.

As we all know, MTF is a key index in the evaluation criteria of an optical system. The index assigned by the telescope system to the MTF drop caused by the change of the pose of the primary lens is 0.98; that is, the MTF drop caused by the change of the pose of the primary lens is less than 2%. Optical analyses show that the decentration of the primary lens is most sensitive to the telescope optical system. Therefore, a tolerance of 1% is assigned to decentration for MTF drop, 0.5% for distance, and 0.5% for tilt. Through Monte Carlo simulation, it can be known that in order to ensure that decentration caused MTF drop within 1%, decentration should be controlled within ±0.02 mm. Similarly, the distance should be controlled within ±0.1 mm, and the tilt should be controlled within ±0.02°. According to Shannon’s sampling theorem, their corresponding measurement accuracy requirements should be 1/5 of the control requirements. In order to achieve the requirement that MTF drops not more than 2%, the control requirements and measurement accuracy requirements of the pose of the primary lens are provided in Table 1.

## 3. Principle of Measurement Method

### 3.1. Establishment of Optical Model of Measurement System

**Figure 2:**
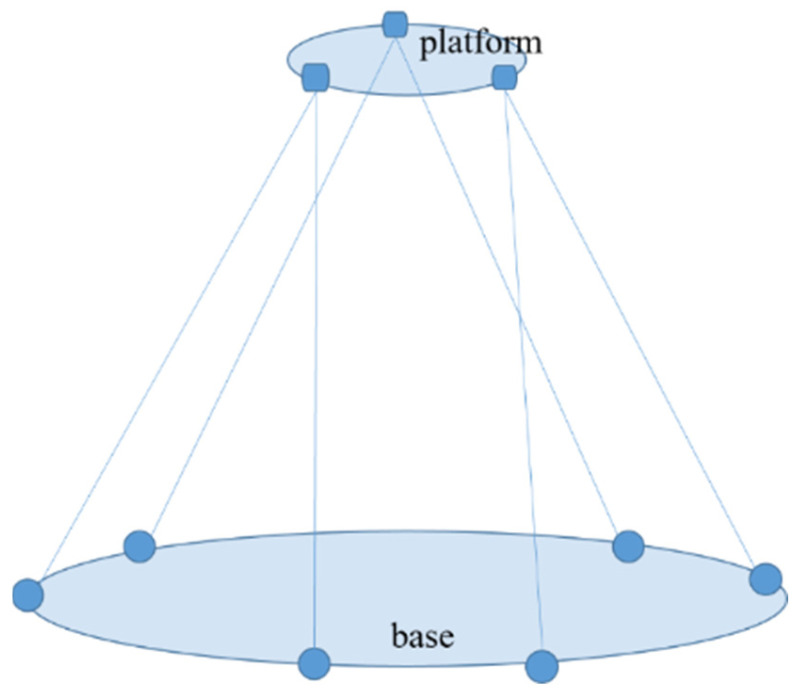
Hexapod geometry Stewart Gough platform.

The method is derived from the principle of the Stewart Gough platform, where the platform and base are connected by six legs at three points on the platform and six points on the base to form a hexapod geometry platform, as shown in Figure 2. The Stewart Gough platform is a mechanical system that uses actuators to drive changes in the length of legs inducing changes in the pose of the platform. For the pose measurement system, the platform legs are the laser optical paths, which constitute the laser optical paths between the primary lens and the relay mirror. In traditional Stewart Gough platforms, forward kinematics is used to map expected changes in platform pose to changes in leg length. For the pose measurement system of the primary lens of the telescope, the laser ranging lengths are known, and the pose of the primary lens must be determined. This is achieved by using an inverse kinematics technique, which generated a sensitivity matrix used to calculate primary lens pose changes from the laser ranging length changes.

**Figure 3:**
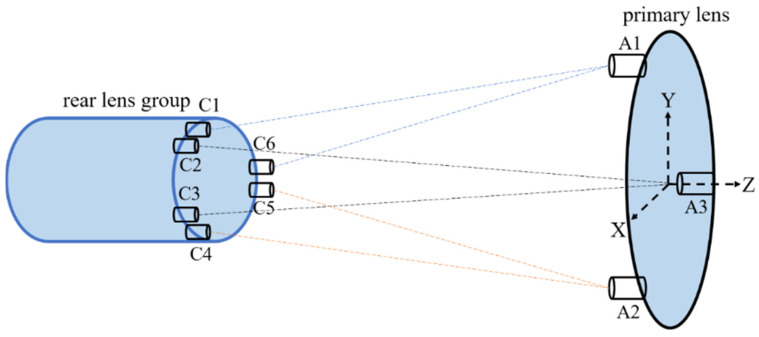
The schematic diagram of the pose measurement principle.

In this method, six laser collimators are fixed on the edge of the relay mirror, and three retroreflectors are fixed on the edge of the primary lens. Each retroreflector provides reflected light for the corresponding two collimators so that six laser distances can be obtained. According to the changes in the six measured distances and the initial coordinates of the collimators and retroreflectors, the pose of the primary lens can be calculated. The schematic diagram of the measurement principle is shown in Figure 3, where C1~C6 are the laser collimators and A1~A3 are the retroreflectors.

### 3.2. Pose Calculation Algorithm

The sensitivity matrix is the basis of the pose calculation technique. The sensitivity matrix defines the sensitivity of the pose changes of the primary lens relative to the relay mirror to changes in the laser length. The change in the laser length is defined as the sensitivity matrix multiplied by the changes in the pose

(1) $$\vec{\Delta l}=S\cdot \vec{p}$$

where *S* is the sensitivity matrix, $\vec{p}$ is the pose vector which includes three translations and three rotations, and $\vec{\Delta l}$ is the laser length change. This is a simple linear algebraic equation, which is the basis of the Stewart Gough platform pose calculation.

The sensitivity matrix is formed using the coordinates data measured by a laser tracker. The laser path direction vectors must be established first. The coordinates of retroreflectors *a_i_*, and the coordinates of collimators *c_i_* are measured. The direction vectors *e_i_* of the laser are created

(2) $$e_{i}=\frac{c_{i}-a_{i}}{\left|c_{i}-a_{i}\right|},(i=1,\ldots ,6)$$

The sensitivity matrix *S* is created by cross-multiplying the coordinates of the laser collimators *c_i_* with the direction vectors. The origin of coordinates is located in the center of the primary lens.

(3) $$S=\left[\begin{array}{cc} c_{1}\times e_{1} & e_{1}\\ \vdots & \vdots \\ c_{6}\times e_{6} & e_{6} \end{array}\right]$$

The sensitivity matrix is a 6 × 6 matrix that defines the sensitivity of the system to the six degrees of freedom of rotation around and translation along the *x*-*, y*-, and *z*-axis of the primary lens.

After the sensitivity matrix is established, we measure six distances from the retroreflectors to the laser collimators *L*0*_i_* in the initial state. Then, the six distances from the retroreflectors to the laser collimators in real-time *L_i_* are measured. And the changes in distance are calculated

(4) $$\Delta l_{i}=L_{i}-L0_{i},(i=1,\ldots ,6)$$

In forward kinematics, the sensitivity matrix is multiplied by the desired pose to determine the changes in path length. The inverse of the sensitivity matrix is needed to calculate the changes in the pose from the changes in laser path length. The changes in the pose of the primary lens relative to the relay mirror $\vec{p}$ are calculated

(5) $$\vec{p}=S^{-1}\cdot \vec{\Delta l}$$

where $S^{-1}$ is the inverse of the sensitivity matrix, $\vec{\Delta l}$ the changes of laser leg length, which can be obtained by two laser rangefinders.

## 4. Experiments

### 4.1. Laser Rangefinders

**Figure 4:**
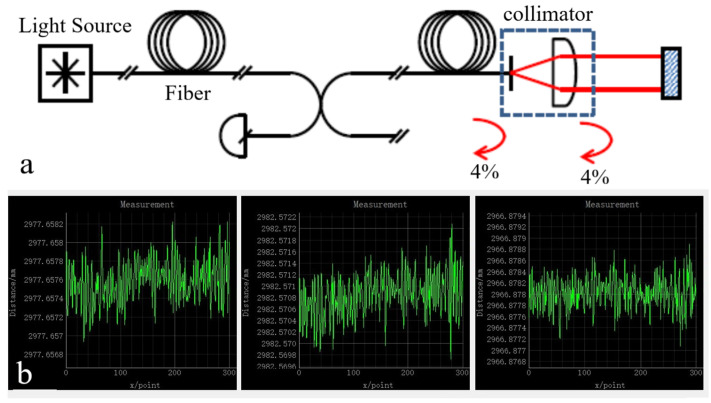
Principle and accuracy test for the laser rangefinder. (**a**) Principle of the laser rangefinder, which is based on Fabry-Perot interference. (**b**) Accuracy test for the laser rangefinder, the laser ranging accuracy is ±0.5 μm.

Laser rangefinders are the key equipment in experiments; the stability of their measured data largely determines the accuracy of experimental results. In addition, they have a long working distance range of up to 50 m, allowing for measurements to be taken over large distances. The laser rangefinders used in the experiment are two sets of IDS3005 laser rangefinders from ATTOCUBE Company (Munich, Germany), and the measurement principle is shown in Figure 4a. One rangefinder weighs less than 2 kg, which makes it a significant advantage for use in space telescopes. With a resolution of 1 pm and a repetition accuracy of 2 nm, one rangefinder has three laser channels and can measure three distances of data simultaneously. When two rangefinders are connected to a computer by a switch, six laser channels can be measured at the same time, which constitutes six laser paths of the measurement system.

IDS3005 laser rangefinder adopts the principle of Fabry-Perot interference [25], which has many advantages: The size of the laser collimators can be very small; the beam spacing can be arranged freely; directly measuring the metal surface; directly measuring the Angle by three beams; allowing a large target Angle deviation; little environmental impact; easy installation and debugging; used in vacuum, low temperature, radiation environment. These advantages make the measurement fairly simple. Each channel consists of a telecom fiber that is coupled to a collimator that transmits a beam of laser to the retroreflector. The backward reflection of the fiber tip is the reference arm of the rangefinder, and the measuring arm is the path through the collimator to the retroreflector and back again. The measured optical path difference (OPD) corresponds to the distance between the fiber tip and the retroreflector.

The measurement accuracy of the laser rangefinder was tested. The test was carried out in a standard laboratory environment, where the retroreflectors were fixed at about 3 m away from the collimators of the laser rangefinder, three channels of one laser rangefinder were measured in 5 min, and the measured distance data were recorded by a data recording software, as shown in Figure 4b. According to the measured data, it was determined that the laser rangefinder is accurate to within ±0.5 μm in such experimental conditions, with little variation between two separate rangefinders. This is consistent with the measurement accuracy data given by the manufacturer. Therefore, the error is acceptable and can meet the experimental requirements. Therefore, we can use these instruments for the pose measurement experiment.

Then, in order to determine the pose measurement accuracy under such laser ranging accuracy, the relationship between the laser ranging accuracy and the pose measurement accuracy was calculated by 10,000 Monte Carlo simulations, and the results are shown in Table 2. This experimental system is highly accurate and precise in terms of rotation around the x and y-axes, rotation around the *z*-axis, and translation along the x-, y-, and z-axes. For example, in the experimental system, when the laser ranging accuracy is ±0.5 μm, the accuracy of rotation around the x and y axes (tilt) Rx/Ry is 0.03″, the accuracy of rotation around the z-axis (roll) Rz is 0.07″, the accuracy of translation along the x and y axes (decentration) is 0.35 μm. The accuracy of translation along the *z*-axis (optical axis direction of the telescope) is 0.15 μm, respectively, indicating highly precise control of the optical axis direction of the telescope. Overall, this experimental system is highly precise and would be ideal for conducting experiments that require highly accurate measurements of position and orientation.

### 4.2. Ground Test

**Figure 5:**
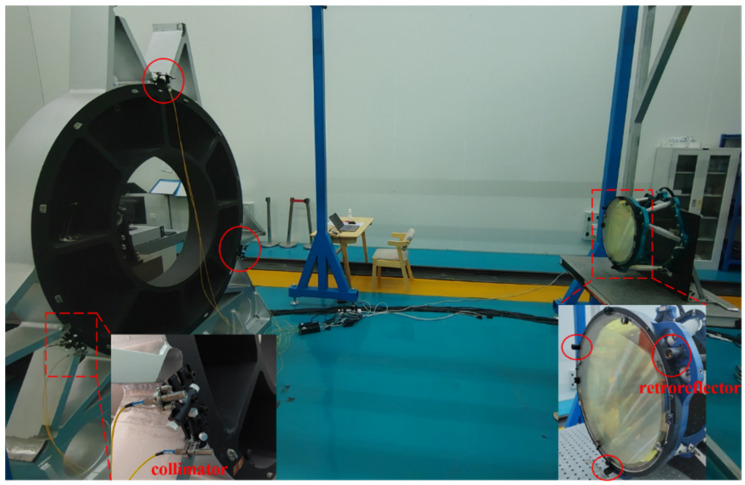
Test system. Three retroreflectors (red circles on the right of Figure 5) were attached to the edge of the frame, and six laser collimators (red circles on the left of Figure 5) were fixed to the edge of the relay mirror to verify the pose measurement method and its accuracy.

In order to verify the pose measurement method and its accuracy, an experimental system was built, as shown in Figure 5. For convenience, a frame was used instead of the primary lens of the telescope and its center was aligned with the center of the relay mirror. At the same time, the frame was fixed to a high-precision hexapod platform to provide an accurate reference pose. Three retroreflectors were attached to the edge of the frame. In addition, six laser collimators were fixed to the edge of the relay mirror and connected to the laser rangefinders by six fibers. Each retroreflector and collimator weigh only about 20 g, which makes the overall measurement system very light and flexible.

After the experimental equipment was installed, the frame was first adjusted to the initial position by the hexapod platform, and the six laser collimators were aligned with the corresponding retroreflectors. Six initial distances were recorded at the initial position, and then the hexapod platform was controlled to drive the frame; the change values of the six new distances were recorded, and the pose can be calculated by the algorithm described above. The pose obtained by this measurement method can be compared with the reference pose of the hexapod platform to verify the accuracy of this measurement system.

**Figure 6:**
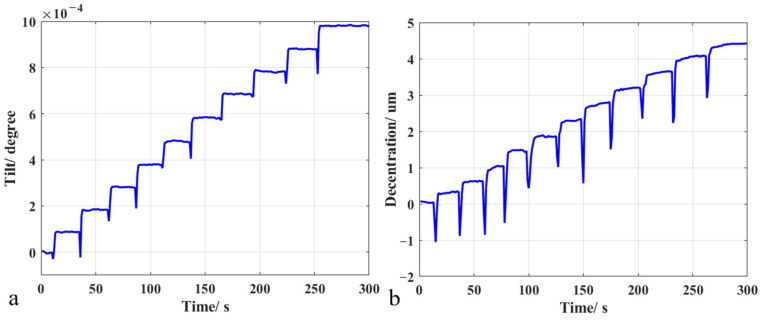
Test results of 1 × 10^−4^ degrees and 0.4 μm step test of the measurement system. (**a**) The tilt error of the measurement system is 2 × 10^−5^ degrees, (**b**) The decentration error of the measurement system is 0.2 μm.

To determine the accuracy of rotation and translation of the pose measurement method, the hexapod platform was driven to rotate around the *x*-axis for step movement every 1 × 10^−4^ degree and move along the *x*-axis for step movement every 0.4 μm. The pose data were measured once a second in 5 min. Figure 6 shows the test results of 1 × 10^−4^ degrees and 0.4 μm step test of the measurement system, respectively. The error of rotation is 2 × 10^−5^ degrees (0.072 arcsec), and the error of translation is 0.2 μm. The spikes on the curve are caused by the fact that the hexapod platform has not been stabilized during the motion. The test results of rotation around the *y*/*z*-axis and translation along the *y*/*z*-axis are almost the same. The experimental results show that the accuracy of the measurement system for tilt reaches 1 × 10^−5^ degree level, and the measuring accuracy for decentration reaches 100 nanometers level, both of which are much better than the measurement requirements. The measurement and calculation were carried out on an ordinary laptop computer, and the single calculation time is 5~10 milliseconds, which achieves the purpose of high-precision real-time measurement.

### 4.3. Error Analysis

During the experiment, there were some errors introduced by the environment and other factors. The analysis shows that the errors are about tens of nanometers. These error sources can be divided into three categories, namely, random errors, systematic errors and environmental errors.

The random errors include detector noise, optical noise, reading error, etc. For the measurement system, the typical value of these noises is about a few nanometers and can be further reduced by averaging the distance data. Systematic errors include some nonlinear periodic errors, which may be caused by optical crosstalk in the light source and electrical crosstalk between reference channels. These errors should be controlled by the design of the rangefinder system, which has little influence on the measurement results. Finally, the environmental errors are mainly caused by ambient temperature and humidity changes and air disturbance, which affect the stability of ranging data. Fortunately, we can reduce these errors caused by environmental factors through the environmental information compensation module of the laser rangefinder and finally control the errors caused by environmental factors within tens of nanometers.

Overall, the errors introduced by the environment and other factors can be mitigated through careful design of the rangefinder system and the use of compensation modules to reduce the impact of environmental factors. By minimizing these errors, the accuracy and reliability of the laser rangefinder can be greatly improved, making it an essential tool for a wide range of applications, from surveying and mapping to industrial positioning and robotics.

## 5. Conclusions

In this paper, a reasonable solution for pose measurement of a long focal length optical telescope in orbit is proposed. Considering the simplicity and accuracy, a measurement system is provided to realize high-precision real-time measurement. The step test results show that the rotation error of the measurement system is 2 × 10^−5^ degrees (0.072 arcsecs), the translation error is 0.2 μm, and the single calculation time is 5~10 milliseconds, achieving the purpose of high-precision real-time measurement. This method has the advantages of simple structure, flexible installation, lightweight, non-contact, high-precision and real-time, which lays a technical foundation for the pose measurement of the primary lens of the space telescope. This measurement method also can be applied to other optical systems and other related systems.

## Figures and Tables

**Table 1:** Influence of each degree of freedom of pose on imaging and measurement accuracy requirements.

Pose Tolerance of Primary Lens	MTF Influence	Tolerance	Measurement Requirements
Distance (translation along the *z*-axis)	0.5%	±0.1 mm	20 μm
Decentration (translation along the *x*/*y* axis)	1%	±0.02 mm	4 μm
Tilt (rotation around *x*/*y* axis)	0.5%	±0.02°	0.004°

**Table 2:** Analysis accuracy (σ).

Degree of Freedom	Accuracy
Rx,y	0.03″
Rz	0.07″
x,y	0.35 μm
z	0.15 μm

## Data Availability

Data underlying the results presented in this paper are not publicly available at this time but may be obtained from the authors upon reasonable request.

## References

[1] Arbabi E., Arbabi A., Kamali S.M., Horie Y., Faraon A. (2016). High efficiency double-wavelength dielectric metasurface lenses with dichroic birefringent meta-atoms. Opt. Express.

[2] Genevet P., Capasso F., Aieta F., Khorasaninejad M., Devlin R. (2017). Recent advances in planar optics: From plasmonic to dielectric metasurfaces. Optica.

[3] Zhang Y., Jiao J., Wang B., Jin J., Su Y. (2015). Transmissive diffractive membrane optic for large aperture lightweight optical telescope. Proc. SPIE.

[4] Liu D., Wang L., Yang W., Wu S., Fan B., Wu F. (2018). Stray light characteristics of the diffractive telescope system. Opt. Eng..

[5] MacEwen A.H., Breckinridge J.B. (2013). Large diffractive/refractive apertures for space and airborne telescopes. Proc. SPIE.

[6] Britten A.J., Dixit N.S., DeBruyckere M., Steadfast D., Hackett J., Farmer B., Poe G., Patrick B., Atcheson D.P., Domber L.J. (2014). Large-aperture fast multilevel Fresnel zone lenses in glass and ultrathin polymer films for visible and near-infrared imaging applications. Appl. Opt..

[7] Atcheson P., Stewart C., Domber J., Whiteaker K., Cole J., Spuhler P., Seltzer A., Britten A.J., Dixit N.S., Farmer B. (2012). MOIRE-Initial Demonstration of a Transmissive Diffractive Membrane Optic for Large Lightweight Optical Telescopes. Proc. SPIE.

[8] Rahlves M., Rezem M., Boroz K., Schlangen S., Reithmeier E., Roth B. (2015). Flexible, fast, and low-cost production process for polymer based diffractive optics. Opt. Express.

[9] Zhao B., Shi W., Zhang J., Zhang M., Qi X., Li J., Li F., Tan J. (2018). Six Degrees of Freedom Displacement Measurement System for Wafer Stage Composed of Hall Sensors. Sensors.

[10] Fan K.C., Chen M.J., Huang W.M. (1998). A Six-Degree-Of-Freedom Measurement System for The Motion Accuracy of Linear Stages. Int. J. Mach. Tools Manufact..

[11] Hu Y., Miyashita L., Watanabe Y., Ishikawa M. (2017). Robust 6-DOF motion sensing for an arbitrary rigid body by multi-view laser Doppler measurements. Opt. Express.

[12] Henselmans R., Nijkerk D., Lemmen M., Rijnveld N., Kamphues F. (2012). Design, analysis, and testing of the optical tube assemblies for the ESO VLT four laser guide star facility. Proc. SPIE.

[13] Kim D.W., Esparza M., Quach H., Rodriguez S., Kang H., Feng Y., Choi H. (2021). Optical Technology for Future Telescopes. Proc. SPIE.

[14] Salvadé Y., Schuhler N., Lévêque S., Floch L.S. (2008). High-accuracy absolute distance measurement using frequency comb referenced multiwavelength source. Appl. Opt..

[15] Jia X., Liu Z., Tao L., Deng Z. (2017). Frequency-scanning interferometry using a time-varying Kalman filter for dynamic tracking measurements. Opt. Express.

[16] Yan L., Xie J., Chen B., Lou Y., Zhang S. (2021). Absolute distance measurement using laser interferometric wavelength leverage with a dynamic-sideband-locked synthetic wavelength generation. Opt. Express.

[17] Han S., Kim Y., Kim S. (2015). Parallel determination of absolute distances to multiple targets by time-of-flight measurement using femtosecond light pulses. Opt. Express.

[18] Gao G., Wang L., Shi H., Liu D., Fan B., Guan C. (2020). Facile large-area uniform photolithography of membrane diffractive lens based on vacuum assisted self contact method. Sci. Rep..

[19] Dale J., Hughes B., Lancaster J.A., Lewis J.A., Reichold J.A., Warden S.M. (2014). Multi-channel absolute distance measurement system with sub ppm-accuracy and 20m range using frequency scanning interferometry and gas absorption cells. Opt. Express.

[20] Zhu K., Guo B., Lu Y., Zhang S., Tan Y. (2017). Single-spot two-dimensional displacement measurement based on self-mixing interferometry. Optica.

[21] Lu C., Liu G., Liu B., Chen F., Gan Y. (2016). Absolute distance measurement system with micron-grade measurement uncertainty and 24m range using frequency scanning interferometry with compensation of environmental vibration. Opt. Express.

[22] Liu C.S., Hsu H.C., Lin Y.X. (2020). Design of a six-degree-of-freedom geometric errors measurement system for a rotary axis of a machine tool. Opt. Lasers Eng..

[23] Friedrich C., Kauschinger B., Ihlenfeldt S. (2019). Spatial force measurement using a rigid hexapod-based end-effector with structure-integrated force sensors in a hexapod machine tool. Measurement.

[24] Gao G., Shi H., Wang L., Liu D., Wang J., Du J., Bian J., Fan B., Yang H. (2022). Large Aperture High Diffraction Efficiency Off-axis Fresnel Lens Fabrication and Analysis. Opt. Express.

[25] Thurner K., Quacquarelli F.P., Braun P.P., Savio C.D., Karrai K. (2015). Fiber-based distance sensing interferometry. Appl. Opt..

